# Nutrition to Optimise Human Health—How to Obtain Physiological Substantiation?

**DOI:** 10.3390/nu13072155

**Published:** 2021-06-23

**Authors:** Renger F. Witkamp

**Affiliations:** Division of Human Nutrition and Health, Wageningen University & Research (WUR), 6700 AA Wageningen, The Netherlands; renger.witkamp@wur.nl

**Keywords:** health claims, EFSA, biomarkers, nutrition, homeostasis, resilience

## Abstract

Demonstrating in an unambiguous manner that a diet, let alone a single product, ‘optimizes’ health, presents an enormous challenge. The least complicated is when the starting situation is clearly suboptimal, like with nutritional deficiencies, malnutrition, unfavourable lifestyle, or due to disease or ageing. Here, desired improvements and intervention strategies may to some extent be clear. However, even then situations require approaches that take into account interactions between nutrients and other factors, complex dose-effect relationships etc. More challenging is to substantiate that a diet or a specific product optimizes health in the general population, which comes down to achieve perceived, ‘non-medical’ or future health benefits in predominantly healthy persons. Presumed underlying mechanisms involve effects of non-nutritional components with subtle and slowly occurring physiological effects that may be difficult to translate into measurable outcomes. Most promising strategies combine classical physiological concepts with those of ‘multi-omics’ and systems biology. Resilience-the ability to maintain or regain homeostasis in response to stressors-is often used as proxy for a particular health domain. Next to this, quantifying health requires personalized strategies, measurements preferably carried out remotely, real-time and in a normal living environment, and experimental designs other than randomized controlled trials (RCTs), for example N-of-1 trials.

## 1. Introduction

In addition to being tasty, nutritious and safe, the principal requirement for our diet is that it should provide essential nutrients that enable optimal development and functioning in daily life. From a biological perspective, this implies that our diet should sustain and, when needed and possible, improve the ability to adapt to the constantly changing physical, social and psychological challenges to which we are exposed. In other words, nutrition is crucial to help us reach our full health potential, in the light of factors such as genetic make-up, age, diseases, social and environmental factors and demands. This ‘ability to adapt’ as a paradigm for optimal health was coined by French physiologists such as Claude Bernard (1813–1878) and later Georges Canguilhem (1904–1995). As Canguilhem also noted, ‘health’ should neither be considered a state of ‘normality’, nor a ‘permanent’ state [1]. The latter certainly also applies to the relationship between nutrition and health when we consider the importance of nutrition for normal development, resistance to diseases, recovery and healthy ageing.

Optimising the current and, when possible also future- ability to adapt to our physical and social environment, often operationalised as capacity to deal with stressors and other perturbating factors, is increasingly recognised and used as a practical concept to establish positive health effects of foods and diets. As will be further elaborated on, these principles and their application are fuelled by the developments in systems biology, ‘omics’ technologies, big data analysis and, increasingly, artificial intelligence.

The capacity to maintain or regain homeostasis is a basic biological principle to preserve optimal interaction with the environment and, ultimately, survive and achieve evolutionary success. Homeostasis is continuously stretched by perturbations and stressors of all different sorts. This requires that our biological systems possess resilience, the power to return to a state of homeostasis after being challenged by perturbation or stress, which is either the original state or another balanced equilibrium.

When a person is in an overall healthy and stable state, the diet should, within a certain time-window, provide the elementary molecular building blocks to literally feed and maintain the dynamic biological equilibria in the body. Depending on demand, developmental stage and environmental factors, this allows a certain degree of time variation in intake, depending on the nutrients of concern. For example, it is relevant to what extent storage can take place in the body. Therefore, in general, it is foremost a ‘balanced diet’ and not a matter of single nutrients or food products that are important to secure homeostasis and allow optimal development. However, it is clear that several situations exist where this reasoning does not hold. First, deficiencies can arise because dietary patterns are often not sufficient, whether or not in relation to specific needs, for example resulting from ageing, strenuous physical exercise or disease. In certain groups, including elderly, overt malnutrition due to psycho-social or economic reasons is also common. Other common causes of deficiencies include medication, malabsorption, etc. Second, and to some extent related, is that our lifeline of homeostasis and health is not horizontal and that loss of health and physiological changes over time are inevitable. In conjunction with this, homeostatic resilience will decline with, for example, ageing or following illness. This may, temporarily or more permanently, require specific dietary adaptations, including the use of specific dietary products. A third, most elusive factor arises from the gap that exists between our biologically optimal way of living and the current situation in which we find ourselves. It is conceivable that these optimal living conditions will never have existed in human history and that we are even closer to them than ever before, as shown, for example, by the increased life expectancy. However, it is also clear that unhealthy dietary habits continue to play a major role in the stagnation of healthy life years [2]. Different food patterns are known, for example, the Okinawan [3] or the Mediterranean diet [4,5], that provide health benefits which are at least partly independent of their energy provision and relative content of important nutrients. Such diets are known to contain components that provide specific health benefits. Examples include non-digestible fibres and a large variety of bio-active molecules produced by microorganisms and plants, the latter sometimes referred to as phytochemicals. During the last decades, knowledge of these compounds, their mechanisms of action and cause-effect relationships has increased considerably. At the same time, these insights are often derived from in vitro or animal studies only, in which usually only individual substances have been tested.

This means that translation of their effects, including those of combinations of compounds, into concrete, perceivable health benefits, such as an improved capacity to deal with health threats that lie in the future, often remains an enormous challenge. It is particularly about these types of health effects and their substantiation where the scientific and regulatory debate is most intense. Clearly, many health products, in particular food supplements, contain substances that have no history as dietary components and originate from herbal remedies instead. In several cases, their claims, whether approved or not, and despite formal regulations, often seem more pharma-like to consumers. It is also difficult to explain to consumers that some natural products, like St. John’s Wort can apparently be the basis of both supplements and medicines. The European regulatory framework on health claims draws a clear line when it comes to disease claims [6]. Regulation (EC) No 1924/2006, on nutrition and health claims made on foods only allows claims referring to the reduction of a risk factor for disease, and not to diseases themselves. In line with this, Regulation (EU) No 1169/2011 states that ‘food information to consumers shall not attribute to any food the property of preventing, treating or curing a human disease, nor refer to such properties’.

Science is increasingly able to demonstrate ‘beneficial physiological effects’, as regulators have formulated it, of diets and foods. Moreover, considerable progress has been made when it comes to understanding the biology of health and its transition to disease. Next to this, practical possibilities of ‘food as medicine’, achieving health gains for patients, are becoming increasingly important. However, as will also be described in this review, the question how nutrition can optimize health in a measurable way cannot be answered in a general sense. This very much depends on the starting situation, the relationship between intervention and expected changes, and the intended goals. However, even with using approaches and insights as described in this review, it often remains more difficult to substantiate that a diet, let alone a single product ‘optimizes health’ than to demonstrate that a drug has beneficial effects with a specific disease.

## 2. Concepts of Maintaining, Improved or Declining Health

### 2.1. How to Define Health?

The question of what health is and how to optimize, improve or even predict its future development, has intrigued generations of scientists [7]. Health refers to a dynamic and multidimensional state and is to a large extent subjectively experienced [3,8]. This has also been recognized in the well-known WHO definition (1946) of health as ‘a state of complete physical, mental and social well-being, and not merely the absence of disease or infirmity’ [9]. However, discussions on the definition of health continue to this day. For example, some are of the opinion that most current definitions are too medical and (or) too much focused on the present, while not taking into account elements of future development and the ability to achieve personal goals [10,11]. A direction that has gained popularity in particular in relation to nutrition builds further on the principles of physiological resilience and robustness, also referred to as ‘the ability to adapt’ [1,12,13]. Huber and colleagues [14] have elaborated this into their concept of ‘positive health’ which encompasses a range of indicators categorized into six domains (including mental functions and perception, the spiritual/existential domain, societal participation etc.). This concept is also receiving criticism, for example that it relies too much on reactive instead of proactive actions for health [10] or that measuring ‘positive health’ is too complicated and that better refinement of the conceptualization is needed [15].

**Figure 1:**
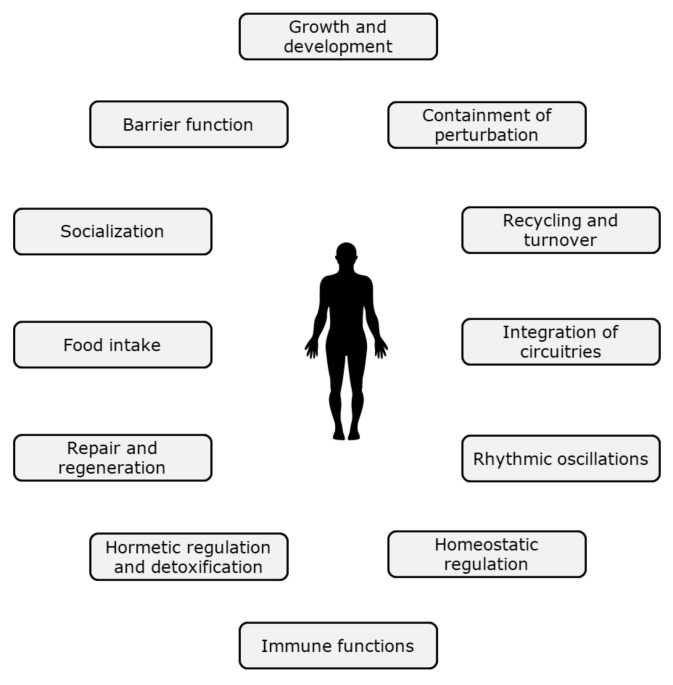
Examples of biological hallmarks (mechanisms) important for health maintenance [16,17].

However, in view of the complexity of nutritional biology and the extent to which we currently understand cause effect-relationships, concepts based on ‘the ability to adapt’ or resilience are offering practical strategies to move forward in this context, allowing experimental physiological- and also psychological approaches to measure health and health optimisation. Such concepts are also getting increasingly mechanistic support from fundamental biology. For example, Ayres [16] proposes a conceptual framework for the ‘biology of physiological health’. According to this idea, organisms have evolved adaptive mechanisms that actively promote the healthy state of an individual. These health mechanisms are generally distinct from those that drive disease. A similar approach is taken in the very recent paper by López-Otín and Kroemer [17], who describe a combination of ‘biological causes’ or ‘hallmarks’ of health. These comprise features of spatial compartmentalization (integrity of barriers and containment of local perturbations), maintenance of homeostasis over time (recycling and turnover, integration of circuitries, and rhythmic oscillations), and an array of adequate responses to stress (homeostatic resilience, hormetic regulation, repair and regeneration). Both concepts [16,17] are schematically combined in Figure 1.

As will also be further elaborated on in Section 5, the use of the physiological health concept has important consequences for the choice of biomarkers. These are not only different from those for disease, but often also more complex and dynamic. From the same principles it will also be clear that, except in case of simple deficiencies of for example of one specific vitamin, there is usually no simple causal relationship between a nutrient and health or clinical endpoint, let alone a simple dose-effect relationship. Instead, as it will be discussed in Section 3, many micronutrients show U-shaped dose-response curves, and several situations are known in which the health outcomes of providing one nutrient can be dependent on the status of one or more others.

### 2.2. From Normality to Response to Stressors as Proxy for Health

Organisms are continuously exposed to different forms of stress, for example physical, psychological, or metabolic stress. During recent years much has been learned about the biology of stress and mechanisms that mediate a return to homeostasis (see for example [18,19]). Stressors induce a plethora of coordinated and dynamic processes aimed at protection, maintenance of homeostasis, adaptation and restoration. Recent research suggests that organisms possess an evolutionarily conserved intracellular signalling network, referred to as integrated stress response (ISR), that helps cells, tissues, and organisms to adapt to a variable environment and maintain health [18]. The eventual course and outcome of exposure to stress is depending on various factors, related to the individual, the environment, as well as the degree and duration of the stressor(s). Hence, stress and the ability to deal with it are important determinants of disease, ageing etc. At the same time, the response to a stressor can provide information about the health status of a person. This principle is well-known from clinical practice, where for example an ECG and other physiological parameters are recorded during and after an exercise challenge, or blood glucose measured during an oral glucose tolerance test. This concept is also increasingly used in research settings, including for nutrition research, which is elaborated on in more detail in Section 5.1. The fact that a response to a stressor in itself can depend on the environment, time of the day etc. entail that such measurements will increasingly be made outside the laboratory, in the daily environment and in a continuous manner. This is facilitated by the rapid developments in the field of continuous biomonitoring, the emergence of a multitude of equipment (wearables) and the fast evolution in data management.

### 2.3. Loss of Health and Development of Disease

**Figure 2:**
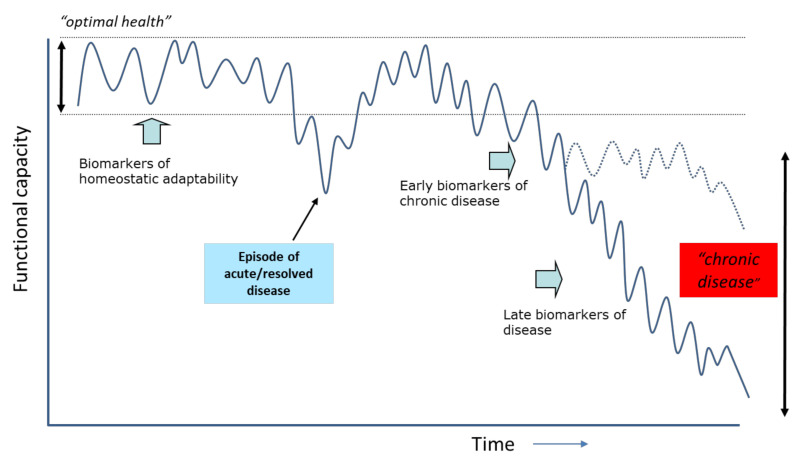
Schematic representation of the concept of homeostasis and loss of health as a function of time. Organisms maintain homeostasis as long as possible by dynamic responses of health mechanisms. Chronic disease is seen as loss of equilibrium. A disease process can either further deteriorate or stabilize at a new homeostatic state.

Although exceptions are obvious, such as with trauma, acute infections, etc., loss of health and development of disease does often not occur overnight. In line with this, the borders between health and disease are generally blurred. In this context, the onset of chronic disease can be considered a consequence of impaired and ultimately the loss of adaptive processes and flexibility of components of our biological system [20]. In many situations, for example with metabolic syndrome, there exists a relatively long period during which the derailment of homeostatic equilibria would be to some extent reversible. This has consequences for the terms ‘normality’ and ‘healthy’ in medicine and biology [21,22]. Also Canguilhem proposes to distinguish the ‘normal’ and the ‘pathological’, instead of ‘health’ and ‘disease’ [1]. When the situation of imbalance between adaptive responses and the effects of stressors or disrupted processes continues, irreversible damage or pathology eventually develops. When this is not adequately managed, a disease process can either further deteriorate, or stabilise at a new homeostatic equilibrium. This concept is schematically illustrated in Figure 2. As depicted, biomarkers and clinical symptoms also change during this evolution to pathology, from those reflecting the ‘normal’ (healthy) state to biomarkers that reflect irreversible disease processes.

### 2.4. Effects of Nutrition to Optimise Health—Differences in Starting Points and Goals

There is no single strategy when it comes to optimising health by nutrition, as starting points, processes and hence roads to improvement can differ considerably. In the next sections, three different starting situations will be distinguished:

#### 2.4.1. Inadequate Nutrition (Section 3)

Here, the starting point is a situation of sub-optimal health that is at least to some extent attributable to insufficient intake and (or) status of specific micronutrients. The underlying causes can be diverse and the number of nutrients of concern might range between one and several. Although in several cases the situation can be improved in a relatively simple manner, there are also pitfalls to be taken into account. These include interdependencies between nutrients, confounders and ‘bystander’ effects, underlying causes that are not resolved etc.

#### 2.4.2. Modulating Suboptimal Health (Section 4)

This is related to an unhealthy lifestyle, ageing, recovery from disease, etc. In many countries, obesity and pre-diabetes are highly prevalent in the general population. Although these conditions may be associated with specific diseases and disorders including diabetes, cardiovascular diseases and dementia, there is often room and a demand for more generic health optimisation. There is often also at least some insight into the relationships between cause, intervention and expected measurable results. Going even further are ‘food as medicine’ strategies which are currently increasingly seen. In this context, optimising health not only comprises lifestyle interventions to improve general health and well-being of patients, but also nutritional strategies to stabilise or even ‘reverse’ the disease process itself [23]. Here too, however, there are several pitfalls that must be taken into account. Long term solutions can be disappointing and require rigorous adaptations of eating habits. As a consequence, quick wins do not exist and single product solutions usually only have short-term and marginal value.

#### 2.4.3. Optimising Future Health (Section 5) in Apparently Healthy (‘Normal’) Persons

Both from a physiological as well as a regulatory point of view this is the most challenging category. Although there may be some overlap with the previous category, including with respect to target groups, the emphasis lies here on effects that go beyond the nutritional value of food components. In addition, the health domains are much wider than typically related to nutrition and metabolism. Instead, they focus on ‘improving physiological functioning’ of processes and systems including the immune system, bone and joints, sleep, well-being etc. The erratic transitions between normal functioning, abnormal functioning and explicit health complaints often create complications, both scientifically and regulatory. The idea is that physiological functioning and resilience are optimised in a more pro-active manner, resulting in potential health benefits that may in part be beneficial for future situations. Needless to say that pitfalls are manifold, the most relevant being caused by the need to measure subtle physiological effects and translate them to actual and perceivable benefits.

## 3. Correcting Inadequate Nutrition and Deficiencies

**Figure 3:**
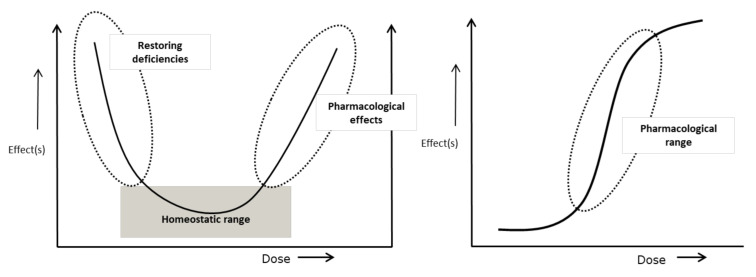
In contrast to most pharmacological compounds (right panel) which usually display sigmoid dose-effect behaviour, most micronutrients often show U-shaped curves. Symptoms of deficiency may be solved by adequate supply (when there are no other limiting factors). When levels are above the optimal (homeostatic) range, effects (non-nutritional or pharmacological) may occur that are often no extrapolation of their biological effects.

The finding that single nutrients and their deficiencies could be linked to specific symptoms and disorders is at the basis of the development of nutrition science as a discipline during the early 20th century [3]. Since that time, several examples of clinically relevant single nutrient deficiencies have become known, and their resolution can prevent serious and sometimes permanent physical or cognitive impairments, or even death. Even today, micronutrient deficiencies in particular, due to poor diets are common and they contribute to major health problems world-wide [24,25]. Furthermore, several risk factors for micronutrient deficiencies are known, including age, use of multiple or specific medication, bariatric surgery, regular strenuous exercise, lack of sun exposure, disease, adherence to specific diets etc. [26,27,28,29,30,31,32]. Next to micronutrients, consumption of adequate amounts of protein also merits attention in certain groups, such as elderly and chronically diseased [23] and as global health issue [33]. Although the case of optimizing health by nutrition may seem rather obvious here, there are still a number of caveats and pitfalls that need to be considered. First of all, it is important to establish whenever possible the underlying cause of the observed deficiencies and to take into account that insufficient nutrient intake or uptake, or an inadequate status do often not occur in isolation. Furthermore, nutrients are involved in several interdependent molecular networks which means that both their deficiencies and their correction may depend on the status of others [34]. These and other factors mean that nutrients generally show U-shaped concentration-effect behaviour, which is in contrast to most medicines where often a sigmoid dose-response curve is observed (Figure 3).

As a consequence, there usually exists an optimum status with a certain bandwidth. Nutrients are often involved in different processes corresponding to different health endpoints with sometimes different optimums. This may also lead to changing viewpoints with time. An example is vitamin D, where adequate plasma levels of the marker metabolite 25(OH)D had originally been estimated based on the role of vitamin D in bone health. However, more recent insights into the significance of vitamin D in relation to other processes, including the immune system and muscle functioning, refuelled the discussions about what optimal levels are, which continues to this day [35,36].

Due to the fact that micronutrients are involved in different processes and may additionally have non-nutritional, pharmacological effects, their pattern of (side-) effects in the descending and ascending part of the dose-response curve are likely to be different as well, making dose-effect extrapolations impossible in these situations. An example is ascorbic acid, vitamin C, which may be administered, including parenterally, at pharmacological doses several folds exceeding the Recommended Dietary Allowance (RDA) [37,38]. The close intertwining of (micro-) nutrients can also lead to a too one-sided focus on correcting single nutrient deficiencies. For example, it has been found that effects of B-vitamins on cognitive functioning in elderly depend on their mutual balance and also on the n-3 fatty acid status [39].

The last but certainly not the least category of pitfalls are related to spurious correlations of which several examples are known in the field of nutrition. For example, measured plasma levels of micronutrients are often misinterpreted since they may be modulated by disease, physical activity or body composition [40,41,42,43]. Taken together, even when correcting apparently simple deficiencies, caution should be exercised and it is important to establish their underlying cause(s).

## 4. Optimising Suboptimal Health with Nutrition

In addition to disorders whose aetiology is directly related to nutrition, such as food allergies or specific metabolic disorders, there are many, in particular chronic, diseases that are at least in part associated with unfavourable eating habits or, more general, an unhealthy lifestyle. The clearest examples are obesity and its cardio-metabolic complications. However, this list of disorders that can at least to some extent be linked to unhealthy lifestyle factors is growing [44,45,46,47]. As has been discussed in Section 2.3, knowledge on the transition processes taking place between a healthy status and clinically manifest disease, typical for many chronic disorders, is rapidly increasing. Next to this, science in the field of ‘health biology’ (Section 2.1) is advancing. Some potential overarching target mechanisms, for example low-grade inflammation, will be further elaborated in Section 5.2. The consequence of these developments is a, to some extent renewed, interest in ‘lifestyle as medicine’, with several positive results being reported, including from nutritional intervention programs. The low hanging fruits are clearly cardio-vascular disease, diabetes type 2 and its comorbidities [48,49,50,51,52,53,54,55,56]. However, there is increasing evidence for causal beneficial effects of nutritional intervention in other diseases as well. Examples include, but are not limited to, depression [57,58], osteoarthritis [59], functional bowel diseases [60], and multiple sclerosis [61]. Next to this, there is increasing insight and interest in nutrition to improve a patient’s overall condition, apart from fighting disease itself. Examples here range from typical clinical nutrition to active lifestyle modification aiming to alleviate symptoms, practical adaptations to improve well-being and prevent co-morbidities etc. Successes of the application of nutrition in case of suboptimal health are also seen at the interface between disease and normal deterioration of health during ageing. In fact, this often involves optimizing the physiological functioning of specific groups in the normal population. As described in Section 2.3 it is also recognized by authorities like the European Food Safety Authority (EFSA) that these situations represent an area where specific health claims are possible [6]. Taken together, it seems conceivable that the number of nutrition-based applications that goes beyond optimising health in the average population is likely to rise.

## 5. Assessing Health Optimisation in Non-Diseased Individuals

### 5.1. The Current Status of Health Claims—The EU as Example

Optimizing health through nutrition in situations where there is no disease (yet) or a direct food-related health problem can be a huge challenge, depending on the grade of scientific evidence needed. Basically, this requires that it is scientifically plausible that a nutritional intervention or a food product in such a situation improves or optimises ‘normal physiology’, resulting in a current or future health benefit. EU Regulation 1924/2006 on nutrition and health claims distinguishes 2 categories of substantiation: based on generally accepted scientific knowledge (‘article 13.1 claims’) or on newly developed scientific data (‘article 13.5 claims’) [62]. Together, these ‘article 13 claims’ are referred to as ‘Function Health Claims’ in the EU, and they are related to:

■ the growth, development and functions of the body;
■ referring to psychological and behavioural functions;
■ slimming or weight-control.

Claims referring to possible future health risks are referred to as ‘Risk Reduction Claims’ (or Article 14(1)(a) claims). It is important to note that only claims on reducing a risk factor in the development of a disease may be made, and not directly on prevention of a disease itself. An example is ‘plant stanol esters have been shown to reduce blood cholesterol. Blood cholesterol is a risk factor in the development of coronary heart disease’ [62]. Beyond the scope of this review are the ‘Article 14(1)(b) claims’ which are claims referring to children’s development.

Although the review takes a physiological perspective and does not intend to provide an in-depth evaluation of the regulatory aspects of nutrition claims, it is illustrative to refer here to the EU Register of Nutrition and Health Claims which lists all authorised and non-authorised health claims (https://ec.europa.eu/food/safety/labelling_nutrition/claims/register/public/?event=search, accessed on 15 April 2021) [63]. The vast majority of the authorised claims to date belong to article 13.1 category. There are currently (April 2021) 14 claims authorised under article 14(1)a and 12 under article 14(1)b. The same Webpage links to a separate list of currently (April 2021) 6 health claims authorized under article 13.5 for which protection of proprietary data is granted (and for which the right of use of the claim is restricted to the benefit of the applicant). From this, it becomes apparent that the majority of the currently allowed health claims fall into the category of ‘generally accepted scientific knowledge’. Of these 229 claims, a vast majority is based on basic biochemistry and physiology, carrying wordings like “contributes to normal metabolism” or “maintenance of normal concentration/functioning”. Furthermore, several micronutrients in the list, with zinc (18 claims) as example, carry multiple claims.

From this the following may be concluded:

(1) For many of these authorized claims, perhaps in particular those of the 13.1 category, it remains very difficult or even unlikely that they can be easily translated into concrete health benefits that can be understood and/or experienced by consumers.
(2) Only a limited proportion of authorized claims seems to be able to meet the demand that their use contributes to measurable health optimization.
(3) The number of physiological studies that have provided novel, convincing scientific evidence is very small. It seems conceivable that this is due to scientific and also economic reasons.

### 5.2. Is Optimisation of Normal Physiology or Risk Factors for Disease via Nutrition Biologically Feasible?

Knowledge from adaptation and training physiology and, more recent findings in the biology of health, resilience and stress add to our understanding of the biological processes that are aimed at maintaining and even improving health through active mechanisms [16,17]. These health mechanisms function at least in part independently from those that drive disease. Ancient principles of ‘training’ are also examples of successful adaptations leading to an improved ability to perform the work or task involved, with effects that often have further-reaching consequences. For example, if dosed properly, physical exercise can increase overall physical and mental fitness. Furthermore, different organs and systems can be ‘trained’, including the cardiovascular and immune system, gastro-intestinal (GI) tract, brain etc. [64,65,66]. Nutrition is among the factors that can induce adaptive responses via different mechanisms. An interesting example are the mechanisms regulated via nuclear factor erythroid 2 (NRF2) and its negative regulator, the E3 ligase adaptor Kelch-like ECH-associated protein 1 (KEAP1). Through activating the transcription of several genes this mechanism plays a major role in the maintenance of redox, metabolic and protein homeostasis, as well as the regulation of inflammation [67,68,69]. NRF2-mediated processes are typical for a phenomenon called hormesis [70]. Hormesis can be regarded as biological overcompensation to the direct and immediate disruptions in cellular homeostasis in response to subthreshold doses of various stressors. Hormesis can enhance resilience without generating any observable phenotypic alterations [69]. Interestingly, several non-nutritional food components, including curcumin, quercetin, ginseng, green tea, and sulforaphane are known to activate NRF2 [69,71]. Next to NRF2, the body possess other hormetic mechanisms, for example mediated by the constitutive androstane receptor (CAR) and pregnane X receptor (PXR), the aryl hydrocarbon receptor (AHR) [72,73] and the sirtuins (SIRT) [23,74]. In addition to stimulating health-promoting processes, dietary components play important roles in modulating and attenuating processes related to deteriorating health without (yet) causing clinical symptoms. One such overarching process is chronically elevated systemic ‘low grade’ inflammation, which is at the basis of many diseases associated with a unfavorable lifestyle [23]. This phenomenon is also often referred to as ‘metaflammation’ [75,76]. Increasing evidence suggests that its involvement goes well beyond metabolic syndrome, as an elevated inflammatory state is also associated with increased risks for depression, cognitive decline, cancer and chronic inflammatory diseases like osteoarthritis, COPD (chronic obstructive pulmonary disease) and IBD (inflammatory bowel disease) [77,78]. Another mechanism worth mentioning here are the effects of nutrition on the intestinal microbiota. Accumulating data underline that nutrition is among the factors modulating our microbiota, which in turn affects general health, well-being and disease risk via multiple mechanisms including those addressed above [79,80,81,82].

**Figure 4:**
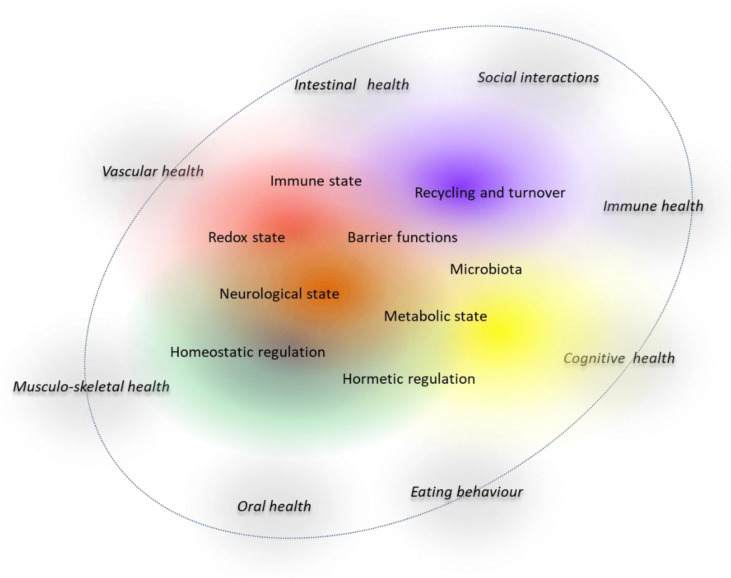
‘Health interactome’ concept to visualise the regulation of different-largely interrelated-health domains by an intertwined set of biological key processes (hallmarks of health) that maintain normal physiology.

Taken together, several lines of evidence underline that nutrition can improve health through processes that are not directly linked to clinical disease. It is important to realise that these effects and adaptations are often confined to specific ‘health domains’ which may be interlinked. For example, an improvement of intestinal barrier by dietary components that activate the AHR can have positive health effects on several organs and tissues [83]. This concept of a ‘health interactome’ is visualized in Figure 4.

Vice versa, nutritional components often display pleiotropic effects. An example are flavonoids, which exert positive effects on vascular health through improving vascular functioning, prothrombotic state, serum lipid profiles, inflammatory- and redox state [84]. To grasp and visualise such effects on multiple processes and (or) health outcomes, concepts of health indexes or a ‘health space’ with different dimensions are also used [85,86]. This provides a “snapshot of health status” composed of different clusters of biomarkers that reflect essential processes such as inflammation, oxidative stress or metabolism.

### 5.3. Biomarkers and Endpoints

It goes without saying that data from human studies are essential to substantiate positive health effects of nutrition. Studies in vitro or in animal models may at best be of supportive value, for example to generate hypotheses or to (further) elucidate mechanisms. The EFSA uses the term pertinent human studies, in which a pertinent study is defined as a study from which scientific conclusions that are relevant to the substantiation of a claim can be drawn [6]. When it comes to substantiation of new health claims, intervention studies are generally considered indispensable. Ideally, a clinical endpoint should be used to establish the effect(s) of the intervention. A clinical endpoint has been defined as ‘a characteristic or variable that reflects how a patient (or consumer) feels, functions or survives’ [87]. However, it is obvious that assessment of clinical endpoints is often not feasible [88,89]. Effects of nutrition are usually subtle and there often exists a long time-period between the introduction of an intervention and the outcome(s). Furthermore, there are several ethical and practical considerations. For this reason, biomarkers are commonly used in practice as proxies or surrogates. A biomarker has been defined as ‘a characteristic that is objectively measured and evaluated as an indicator of normal biological processes, pathogenic processes, or pharmacologic responses to an intervention’ [87]. Examples of biomarkers include blood concentrations of nutrients, LDL-cholesterol and HDL cholesterol, blood pressure (BP), enzyme concentrations, tumour size, genetic variations, and combinations of these measurements [89]. There are many applications of biomarkers and classification schemes of nutritionally relevant biomarker have been proposed [90]. The WHO uses three different classes: biomarkers of exposure, biomarkers of effect and biomarkers of susceptibility [88]. Here, a biomarker of effect is ‘a measurable biochemical, physiological, behavioural or other alteration in an organism that, depending upon the magnitude, can be recognized as associated with an established or possible health impairment or disease’. A biomarker of susceptibility is ‘an indicator of an inherent or acquired ability of an organism to respond to the challenge of exposure to a specific xenobiotic substance’ [88,91]. A more extensive scheme for biomarker classification based on intended use rather than the technology or outcomes was proposed by Gao et al. [90]. Here, six subclasses are suggested: food compound intake biomarkers, food or food component intake biomarkers, dietary pattern biomarkers, food compound status biomarkers, effect biomarkers, and physiological or health state biomarkers.

A surrogate endpoint (or marker) is a biomarker intended (based on epidemiologic, therapeutic, pathophysiologic, or other scientific evidence) to substitute for a clinical endpoint that should predict clinical benefit or harm or lack of both [89]. As introduced in Section 2.3, biomarkers of health are different from those of disease. In addition, multiple, coherent sets of biomarkers, or biomarker profiles [92] are increasingly being applied, which provides a much better insight into the dynamics of physiological processes.

### 5.4. The Nutritional Phenotype and ‘Multi-Omics’ Revolution

In order to better comprehend the complex and subtle effects of nutrition, the concept of the nutritional phenotype has been coined. This term encompasses an integrated set of genetic, proteomic, metabolomic, functional, and behavioural factors that, when measured, form the basis for assessment of human nutritional status [93]. A second development is the application of ’challenge’ or ‘stress’ tests to measure flexibility and resilience as a proxy for health status. As introduced in Section 2.2, the response to a stressor contains information about the capacity to maintain homeostasis and return to normality, which can be considered as indicative for health status [94,95]. Challenge tests measure the response to metabolic, physical, psychological or immunological stressors. Different biochemical, physiological or psychological protocols and endpoints are in use, indicative for specific processes, also referred to as phenotypic flexibility [20,96]. For example, a metabolic challenge test measures the response to a standardized meal or shake that provides a carbohydrate or fat “load’ [95,97]. Physical challenge tests apply exercise as stressor to generate effects on immune function, intestinal permeability, cardiometabolic processes etc. [98,99]. Other examples of challenge tests include the use of vaccination, experimental infection [100] or psychological stress [101,102].

A third, and perhaps ultimately the most important development results from the enormous growth that so-called ‘omics’ technologies have taken. Following the introduction and sharp rise in the use of ‘genomics’ technologies around the 1990s during the last century, a revolution in different ‘omics’ (sub-) technologies has taken place. The addition of “omics” is now used for a comprehensive, or global, assessment of a set of molecules. Well-known and meanwhile almost ‘classical’ examples include transcriptomics, proteomics and metabolomics, but with the developments in different fields of application this list now encompasses many more terms including nutrigenomics, foodomics, glycomics, lipidomics etc. [103,104]. The quantitative combination and integration of these ‘omics’ disciplines is often referred to as ‘multi-omics’. These developments are likely to revolutionize the discovery and understanding of biological processes, including their dynamics in time, metabolic fluxes etc. Although we are still at the beginning, this seems extremely important to really understand what nutrition does to our physiology. At least as important as analysing the molecules themselves are the developments in big-data analysis, statistics and machine learning. Together with information from other channels, including food-frequency questionnaires (FFQs), data on well-being, lifestyle etc. this comes together in the concept of systems biology, which has evolved as a multidisciplinary approach aiming to decipher the complexity of biological systems that starts from the understanding that the networks that form the whole of living organisms are more than the sum of their parts [104,105,106,107].

### 5.5. Methodologies: Examples of Studies and Outcome Measures

Discussions continue about appropriate study designs and outcomes for substantiating health claims in practice. Although it is by no means the intention of the current review to list, let alone to evaluate individual study designs, it is illustrative to provide a number of examples from the literature (Table 1). The table is categorized according to health domain, although it should be noted that this is somewhat arbitrary, as an overlap often exists between these domains in terms of regulation and effects of intervention (see Section 5.2). The table includes data from the guidance documents on the scientific requirements for health claims issued by EFSA [108,109,110,111,112,113,114,115,116], however without consequently following the same categorization. These EFSA documents provide useful background information, definitions, pitfalls and points of attention and are revised from time to time using the experience gained during the evaluations. It should be noted that Table 1 is neither providing an exhaustive nor authoritative overview of possible ‘beneficial physiological effects’ and studies/outcome variables. Regulatory bodies will always follow a case-by-case approach taking into account different factors. In fact, in some of the domains listed below, hardly any claims have received a positive opinion from EFSA, including those using the principles below.

### 5.6. Development in Study Designs and Measurement of Effects

#### 5.6.1. The Need for Alternatives to RCTs

Although only briefly discussed in this article, appropriate study designs are crucial to ultimately reach sound conclusions. The gold standard for clinical research is the double-blind randomized controlled trial (RCT). Blinded RCTs are particularly well suited to generate evidence on drug efficacy and effectiveness, provided that the assignment is concealed, there is proper blinding, intent-to-treat analysis, and a sufficiently large sample size. At the same time, RCTs have also limitations which are mainly related to their limited external validity and their focus on group comparison to estimate treatment effects on population level [144]. Next to this, they are very costly and considered not very efficient. Although RCTs were also adopted in nutrition research, in particular around the ’90s of last century coinciding with the heyday of the ‘functional food’ concept, their design often poses insurmountable problems in this field. Reasons for this are manifold; Effects of nutrients, foods or diets are usually far more subtle and slowly occurring than drugs, the nutrients that people consume are part of complex foods and daily diets which are combined with other foods, problems with blinding and placebos occur, both practical and ethical, there are uncertainties about intake and compliance, interindividual differences are relatively large, and costs very high [53,145,146,147,148,149].

Even results of successful RCTs performed on single components or on single foods often lead to conflicting views on what constitutes a healthy daily diet [150]. For these reasons, the principles of nutritional study designs beyond and in addition to RCTs are being evaluated and discussed in different forums. Obviously, directions in which solutions are sought in relation to the central questions addressed in this review include quasi-experimental designs [151,152,153] and stepped-wedge cluster design [154]. In some cases, for example when investigating the effects of an intensive multicomponent lifestyle programme on a progressive disease like diabetes type 2, single arm trials can be of relevance [155]. Placebo effects are likely to play a role here, but placebo controls are difficult, the normal course of the disease is well known and spontaneous improvements are rare.

Of particular relevance are the renewed interest and developments in the field of so-called N-of-1 trials. N-of-1 trials are designed to measure or observe one person multiple times, with repetition providing statistical power. Next to observational N-of-1 trials, different variations of interventional N-of-1 designs are in use, including multiple cross-over, double-blind, placebo-controlled single person trials [156,157,158,159]. Although the principle of N-of-1 is not new, this design receives a renewed boost following the developments in precision medicine, the importance of inter-individual differences regarding treatment responses, the developments of electronic health information technology (see next section), as well as in statistical approaches to analyse the data. These developments have also attracted the attention of nutrition research where N-of-1 designs offer a number of attractive features [160,161,162]. A review on the use and possibilities of N-of-1 trials specifically for nutrition research that provides an excellent introduction to the topic, was recently published by Potter et al. [161].

#### 5.6.2. Remote and Real-Time Studies

A final development that should not be left unmentioned concerns the rapid developments in the field of remote technologies, sensors and wearables, which has created new opportunities to perform intervention studies in the daily living environment while allowing collection of real-time data via sensors and working with samples like dried blood spots [163,164,165]. In view of the large inter-individual variation and the subtle effects of nutrition, this offers interesting advantages to measure more frequently and over longer periods of time. For example, continuous glucose monitoring is already widely used and oral glucose tolerance tests can now also be performed at home under remote supervision. The use of accelerometers, sleep sensors and devices that monitor various physiological parameters is increasing and it is only a matter of time that nutritional intervention research will mainly take place outside the conventional laboratory setting.

## 6. Conclusions

Clearly, assessing an individual’s ‘degree of health’ is generally much more difficult than making a diagnosis of disease and determining its severity. We still know relatively little about the ‘symptoms of health’, as these will mostly go unnoticed in daily life. However, as described in this narrative, more and more is becoming known about the biology of health. It is also becoming increasingly clear that a combination of active, adaptive and trainable processes are crucial to maintaining and improving health. Different factors in our environment, including various components in the diet, contribute to triggering these mechanisms. Therefore, the question whether nutrition can make a measurable contribution to optimizing our health can be answered in the affirmative.

At the same time, we know that the processes that underly resilience are complex and intertwined. There is no doubt that the effects of nutrition, and certainly those of individual nutrients, are subtle and very slowly manifest. Partly because of this, effects of dietary intervention are to a large extent individually determined, as recent cutting-edge studies have shown [137,163,166].

This places high demands on intervention studies aimed at substantiating health optimization. Since such studies will often be too short to establish positive health outcomes that become manifest in the longer term, we will need to learn more about the biology of health, processes that take place during the fuzzy transition from healthy homeostasis to disease. Scientific consensus about ‘surrogate’ processes that provide meaningful short-term information and the way to assess them, laid down in guidelines when it comes to new claims, is important in this regard. Studies leading to this are preferably carried out by larger (international) collaborations. To meet individual dynamics of nutritional biology and their dependence on other factors including time, such studies will increasingly be conducted in real-life settings and remotely monitored. This allows taking into account daily environmental stressors and effects of actual food intake, combined with data from remote experimental tests. Data obtained will be combined with others such as FFQs, images and other inputs like data stored in a person’s health phenotype database. Although it may seem that there is still a long way to go, developments in the field of sensors, big-data management and machine learning are going extremely fast.

## Figures and Tables

**Table 1:** Examples of study set-ups as per health domain as taken from the literature, including recent EFSA guidance documents.

Health Domain	Study Set-Up
Bone and joint health	Maintenance of bone mass or bone mineral density [111].
	Maintenance of joint function [111].
	Reduced falls and fractures [117].
Cardiovascular health	Beneficial changes in the blood lipid profile [113].
	Reduction in arterial (systolic) blood pressure (SBP) [113].
	Flow mediated dilatation [118].
	Carotid-artery reactivity (CAR) measurement. Effect of a cold pressor test (CPT) [119].
	Arterial stiffness via carotid-to-femoral pulse wave velocity (PWVc-f) [120].
	Arterial stiffness via the augmentation index (AIx) [121].
	Retinal microvascular structure [122].
	Decreased platelet aggregation [123,124].
	Maintenance of normal blood homocysteine concentrations [113].
Cognitive performance, stress, psychological functions and other CNS domains	Improvement or maintenance of cognitive functions [114].
	Improvement of alertness and/or attention [114].
	Improvement of mood/affect [114].
	Psychological stress tests [114,125].
	Anxiety [114].
	Improvement or maintenance of vision [114].
	Improvement of sleep [114].
Gastro-intestinal functionality	Breath hydrogen levels, gas volume assessed by imaging (i.e., MRI). [115,126].
	Transit time, frequency of bowel movements, stool bulk [115,126,127].
	Validated subjective global symptom severity questionnaires [115,126].
	(Changes in) microbiota composition of the gut accompanied by evidence of a beneficial physiological effect or clinical outcome and/or including pathogenic and toxicogenic microorganisms [115,126,128].
	Changes in short chain fatty acid production in the gut [115].
	Changes in digestion or (and) absorption [115,126].
	Changes in structure of intestinal epithelium [115].
	Changes in barrier function, using physical exercise challenge [129,130] or NSAIDs [131].
Immune function and defense against pathogens	Changes in immune markers, e.g., numbers of various lymphoid subpopulations in the Circulation * [115,132].
	Changes in markers of inflammation ^1^ [115,132].
	Metabolic challenge tests ^2^ [132,133].
	Immune-training effects [64].
	An increase in the number of responders to vaccination [115].
	Microbiological data at specific sites [115].
	Improved protection of groups at increased risk [115].
	Beneficial response to allergens [115].
	Response to experimental infections [100,115,132,134].
	LPS challenge [135].
	Response to exercise challenge (indirectly) ^2^ [136].
Metabolic health	Metabolic challenge tests, including mixed meal tests and post-prandial glycemic responses [96,97,116,133,137,138,139,140].
	Protection against oxidative damage (DNA, proteins, Lipids) and DNA breaks [116].
Oral health	Saliva flow or measurement of self-perceived oral dryness by validated questionnaires.
	Maintenance of gum function [111,141].
	Reduction of dental plaque, acid production and/or dental calculus [111,141].
	Maintenance of tooth mineralization [111,141].
	Reduction of oral dryness [111,141].
	Specific colonisation with Streptococcus mutans, decrease of caries [111,141].
Physical performance	Improvement of physical performance (the ability to complete certain physical tasks) at certain (high) intensity and with a certain (long) duration in a physical exercise trial [108,109].
	Physical capacity (exercise time to fatigue at predefined conditions) [109].
	Muscle function (i.e., change in muscle structure) ^3^ [109].
Skin health	Trans epidermal water loss [142].
	Skin Hydration [142].
	Skin Dryness [142].
	Skin Elasticity [142].
	Corneocyte Adhesion [142].
	Oxidative Damage to Lipids [142].
	Protection of the skin against oxidative (including UV-induced) damage [111,142].
	Protection of the skin from UV-induced (other than oxidative) damage [111,142].
Weight management	Appetite ratings [110,143].
	Behavioral assessment (energy intake etc) ^4^ [110,143].
	Body fat (different methods of assessment) and lean body mass (idem) [110,143].
	Bodyweight regain/maintenance (prolonged time period, 6 months) [110,143].

^1^ Provided that these relate to a benefit on specific functions of the body (EFSA). ^2^ Response to such tests may include immunological effects (e.g., on inflammation). ^3^ In the context of a particular type of exercise or physical activity. ^4^ Biochemical markers in support (i.e., cholecystokinin). NSAID: non-steroidal antiinflammatory drug; CNS: central nervous system.
